# Comparison of disinfection by-products formed by preoxidation of sulfamethazine by K_2_FeO_4_ and O_3_ and the influence on cytotoxicity and biological toxicity

**DOI:** 10.3389/fchem.2022.904867

**Published:** 2022-08-19

**Authors:** Siwen Li, Yingzi Lin, Gaoqi Wang, Suiyi Zhu, Gen Liu, Chunyan Shi, Lei Chen

**Affiliations:** ^1^ School of Environment, Northeast Normal University, Changchun, China; ^2^ Science and Technology Innovation Center for Municipal Wastewater Treatment and Water Quality Protection, Northeast Normal University, Changchun, China; ^3^ School of Municipal & Environmental Engineering, Jilin Jianzhu University, Changchun, China; ^4^ Key Laboratory of Songliao Aquatic Environment, Ministry of Education, Jilin Jianzhu University, Changchun, China; ^5^ The University of Kitakyushu, Kitakyushu, Japan

**Keywords:** preoxidation by potassium ferrate, preoxidation by ozone, chlorine disinfection, DBP, toxicity

## Abstract

This study researched the formation of disinfection by-products (DBPs) in sulfamethazine (SMZ) chlorination after preoxidation by K_2_FeO_4_ and O_3_ and the influence of preoxidation on cytotoxicity and biological toxicity. Then, the study emphatically analyzed the influencing factors such as NaClO dosage, pH value, reaction temperature, fulvic acid (FA), and bromide and iodide ions. The results showed that preoxidation by K_2_FeO_4_ effectively inhibited the formation of DBPs of haloketones (HKS) and trihalomethanes (THMs), with an average inhibition rate of over 60%. The formation of DBPs after preoxidation by O_3_ was higher than that by K_2_FeO_4_; preoxidation by K_2_FeO_4_ reduced the influence of NaClO dosage, temperature, and pH value on the production of DBPs after SMZ chlorination. The cytotoxicity and biological toxicity of SMZ chlorination after preoxidation were evaluated and compared by calculating the LC_50_ value of DBPs and the luminescent bacteria method. The results of both calculation methods showed that the toxicity of DBPs after preoxidation by K_2_FeO_4_ was lower than that by O_3_ preoxidation. K_2_FeO_4_ and O_3_ preoxidation improved the SMZ removal efficiency by 8.41 and 10.49%, respectively, and inhibited the formation of most DBPs, but the preoxidation promoted the formation of highly toxic DBPs (HANs). The toxicity of DBPs formed in SMZ chlorination after preoxidation by K_2_FeO_4_ and O_3_ was slightly higher than that of chlorination disinfection alone, but it was still within the safe range. This study provides more new details about the formation and toxicity changes of DBPs in the process of SMZ chlorination after preoxidation.

## Introduction

Drinking water disinfection can effectively prevent the transmission of viruses and other pollutants in water (Baldursson and Karanis, 2011; Mahmood, 2015; Efstratiou et al., 2017; Schijven et al., 2019; Jeon et al., 2022). As a new type of organic pollutant, antibiotics can react with chlorine-containing disinfectants to produce carbon- or nitrogen-containing DBPs (Chao, 2014). So far, fluoroquinolones, sulfonamides, macrolides, and other antibiotics have been detected in drinking water sources in many countries (García-Galán et al., 2010; Shimizu et al., 2013; Molnar et al., 2016; Yha et al., 2021). Some studies believed that antibiotic contamination is one of the factors leading to the spread of antibiotic-resistant bacteria (Baquero et al., 2008; Hu et al., 2021; Dan et al., 2022), and it was also regarded by the World Health Organization as one of the three major threats to human health (Hua and Reckhow, 2013; Pruden et al., 2013).

Sulfonamides (SAs) are considered to be potentially toxic to aquatic organisms and have a high migration ability in water environment media (Chen et al., 2012; Chen et al., 2015; Ou et al., 2015; Sun et al., 2019). They are easy to accumulate in organisms through water and soil to produce resistance genes and finally enter human bodies through food chain, drinking water, respiratory system, and skin contact, etc., causing toxicity and body damage to humans (Kilemade and Mothersill, 2000; García-Galán et al., 2011; Gaffhey et al., 2016).

At present, there are few studies on the removal of SAs by chlorination. There is still no in-depth research on SA removal with the most widely used method for drinking water disinfection being chlorination with HOCl/OCL^−^ (Lizasoain et al., 2018; Tak et al., 2020; Wang et al., 2020). Some studies only described the products produced by the reaction of SAS with chlorine (Dodd and Huang, 2004; Chamberlain and Adams, 2006; Melton and Brown, 2012), and chlorine-containing oxidants are prone to generate chlorinated DBP which may cause mutagenesis, carcinogenesis, and teratogenesis when contacting oxidized aromatic, aniline, and other substances such as humic acid, and it greatly threatened water ecology and biological health. In the drinking water treatment process, advanced oxidation technology is usually used in the preoxidation process in combination with subsequent chlorine-containing disinfectants to reduce the dosage of chlorine-containing disinfectants and the concentration of DBPs in water, thus reducing toxicity.

The study simulated the disinfectant concentration of the conventional disinfection process in a water purification plant and selected the typical sulfonamide antibiotic SMZ as the target pollutant to study the influence of NaClO disinfection alone and disinfection after preoxidation by K_2_FeO_4_ and O_3_ on the formation and change of toxicity of DBPs formed in SMZ chlorination. Key factors such as NaClO dosage, pH value, and temperature were discussed. Since Br^−^ and I^−^ widely exist in natural water, the study also discussed the effect of preoxidation on the formation of Br^−^ DBPs and I^−^ DBPs. At the same time, the cytotoxicity and biological toxicity of DBPs after the reaction were quantitatively analyzed by the LC_50_ value calculation method and luminescent bacteria method.

## Materials and methods

### Drugs and standard reagents

SMZ (purity >99%) was purchased from Aladdin. The NaClO solution was prepared by diluting 5% NaClO solution (Tianjin Guangfu) with ultrapure water. Methanol, formic acid, acetonitrile, and methyl tertbutyl ether detected by high-performance liquid chromatography (HPLC) and gas chromatography (GC) using an electron capture detector (ECD) were of chromatographic grade and purchased from American Tedia Company and Fisher Company. DBP-mixed standard samples were purchased from American AccuStandard Company and Canadian TRC Company. Other analytical grade reagents, together with FA and K_2_FeO_4_, were purchased from Shanghai Macklin Biochemical Co., Ltd. Anhydrous sodium sulfate (Na_2_SO_4_), potassium iodide (KI), and potassium bromide (KBr) were purchased from Beijing Chemical Plant.

### Experimental method

A volume of 100 ml of prepared 5 mg/L SMZ solution was taken from a thermostat water bath (20 ± 0.5°C) and added into a 250-ml conical flask. The pH value was then adjusted to 7. The water samples were dosed with preoxidants K_2_FeO_4_ and O_3_ until the K_2_FeO_4_ concentration (calculated by K_2_FeO_4_) of the water sample reached 2 mg/L, preoxidizing for 30 min, and O_3_ concentration of the water sample (calculated by O_3_) reached 0.4 mg/L. After preoxidation, 5 g/L NaClO solution (calculated by effective chlorine) was added to SMZ water solution until the NaClO concentration of the water sample reached 3 mg/L. A volume of 100 μl of 20 g/L Na_2_SO_3_ was added immediately to the solution to terminate the preoxidation reaction of the water sample after 120 min.

### Measurement of residual chlorine

In this study, the residual chlorine was characterized by the residual amount of sodium sulfite, which was detected using a UV-vis spectrophotometer (HACH dr6000, American Hash Company) and a portable residual chlorine tester (Q-CL501, Shenzhen Qingshijie Technology Co., Ltd.) (Jiang et al., 2015).

### Detection of DBPs

The US EPA 551 microextraction method was used in the measurement of DBPs with liquid–liquid extraction–gas chromatography using anECD gas chromatograph (GC-7890A, Agilent Company). A volume of 50 ml of the water sample was measured and added into a brown glass bottle with a Teflon cap. After adding 20 g of anhydrous sodium sulfate, the bottle was shaken evenly with the cap covered. Then, 3 ml MTBE was added, and the bottle was shaken well and allowed to stand for 3 min. Then, 1 ml of organic in the upper layer was measured, taken in the sample vial, and analyzed using an air inlet chromatograph. The conditions for the reaction were set as follows: capillary column DB-1: 30 m × 0.25 mm×1 μm; detector temperature: 290°C; high-purity nitrogen: ≥99.999%; flow: 20 ml/min constant flow; and tail blowing flow: 5 ml/min.

### Toxicity assessment

Cytotoxicity was assessed using a previously published calculation method by Plewa et al. (Wagner, E. D. et al., 2017). Chinese hamster ovary (CHO) cells were selected as the research object and tested, and the cytotoxicity and genotoxicity of 103 types of DBPs were statistically analyzed by combining with the data from published articles. In the toxicity test, the LC_50_ value was taken as the concentration of each DBP that reduced the growth number of Chinese hamster ovary (CHO) cells by 50%. The molar concentration of each DBP was divided by its corresponding LC_50_ value. The results obtained were used to evaluate the toxicity of DBPs under various influencing factors. In toxicity assessment, it was generally assumed that the toxicity of different DBPs can be cumulated. This method was often used to evaluate the toxicity of detected DBPs (Aleksandra et al., 2017; Liu et al., 2018; Gao et al., 2019).

Biotoxicity was analyzed by the luminescent bacteria method with *Vibrio fischeri* strains of international standard and the LUMIStox 300 instrument. The pH value of the sample was maintained in the range of 6.0–8.5, and the resuscitated luminescent bacteria suspension was used as the stock suspension. According to the measured fluorescence intensity, the correction factor (FKT value) was measured with formula (1), which was used to correct initial I_0_ of all samples before taking it as the reduced fluorescence reference value affected by water:

$${\text{f}}_{\text{kt}}={\text{I}}_{\text{kt}}/{\text{I}}_{\text{0}},$$ (1)

where f_kt_ is the correction factor, I_kt_ is the fluorescence intensity, and I_0_ is the fluorescence intensity of the control sample.

The average f_kt_ value of the control sample is calculated by Eq. 2:

$$I_{\mathrm{ct}}=I_{0}\cdot \bar{f_{\text{kt}}}.$$ (2)

The inhibiting effect of the sample is as follows:

$$H_{t}=\frac{I_{\mathrm{ct}}-I_{\mathrm{Tt}}}{I_{\mathrm{ct}}}\times 100\%.$$ (3)

## Results and discussion

### Influence of preoxidation by K_2_FeO_4_ and O_3_ on the change of DBPs formed in SMZ chlorination

**FIGURE 1:**
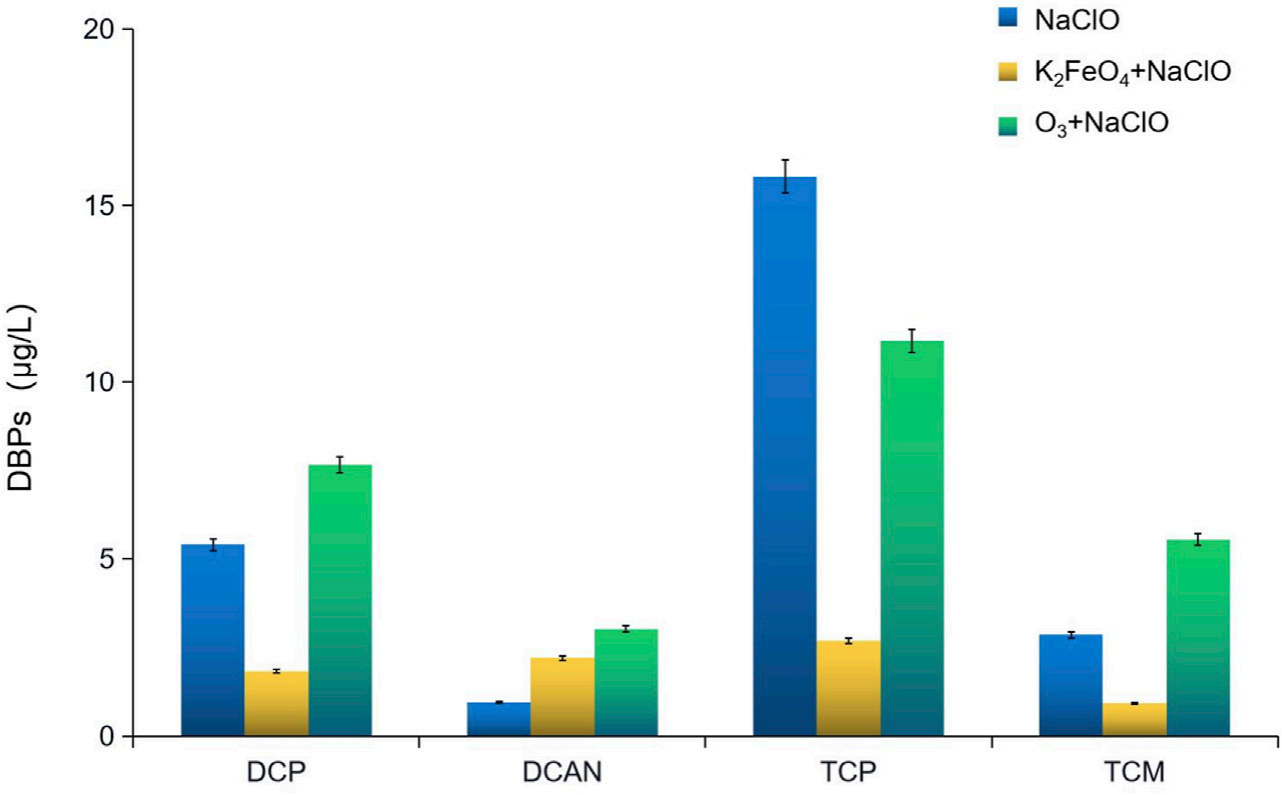
Influence of preoxidation by K_2_FeO_4_ and O_3_ on the change of DBPs formed in SMZ chlorination.

**FIGURE 2:**
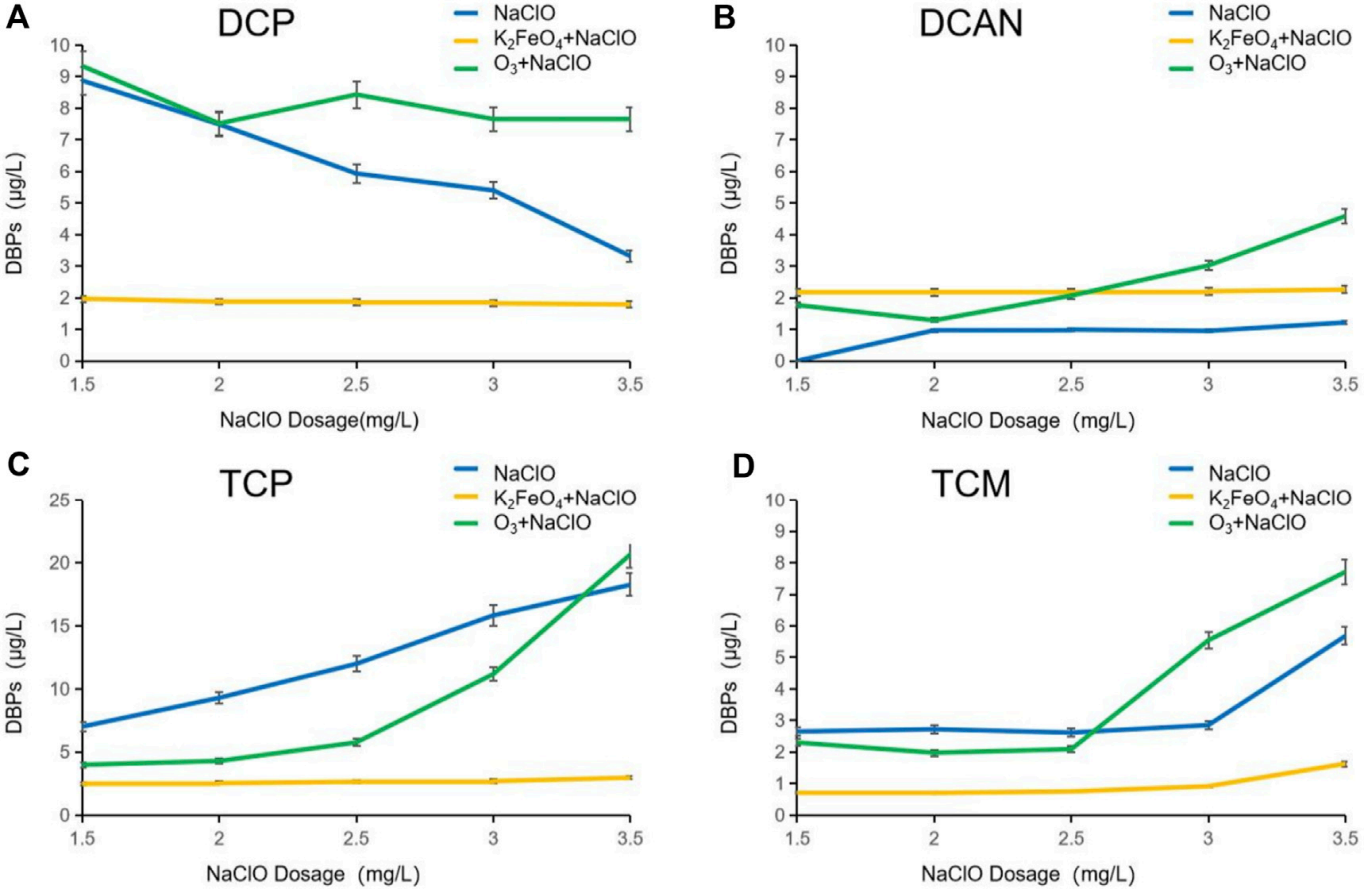
Influence of NaClO dosage on DBPs formed after preoxidation of SMZ by K_2_FeO_4_ and O_3_.

The change of DBPs formed in SMZ chlorination after preoxidation by K_2_FeO_4_ and O_3_ is shown in Figure 1. Preoxidation by K_2_FeO_4_ effectively inhibited the formation of dichloroacetone (DCP), trichloroacetone (TCP), and chloroform (TCM), with inhibition rates of 66.22%, 82.97, and 67.91%, respectively, but promoted the formation of dichloroacetonitrile (DCAN). Preoxidation by O_3_ inhibited the formation of TCP, with an inhibition rate of 29.37%. The change occurred when the dosage of NaClO was increased from 1.5 mg/L to 3.5 mg/L is shown in Figure 2. The formed DBPs in the form of HKs underwent nucleophilic substitution reactions and gradually converted from DCP to TCP. The greater the dosage of NaClO is, the more obvious the conversion is. After preoxidation, the content of DCAN, a type of nitrogen-containing DBP, increased significantly and was higher than that after chlorination alone. The main reason was that preoxidation made the amino group of the target pollutant SMZ fall off easily. When the dosage of NaClO was 2 mg/L, the production of DCAN decreased. The main reason was that the presence of free chlorine led to the instability and self-decomposition of haloacetonitrile, which is consistent with the factors pointed out by Bond et al. (2012). The production of TCM increased continuously with the dosage of NaClO.

### Influence of the pH value on DBPs formed after preoxidation of SMZ by K_2_FeO_4_ and O_3_

**FIGURE 3:**
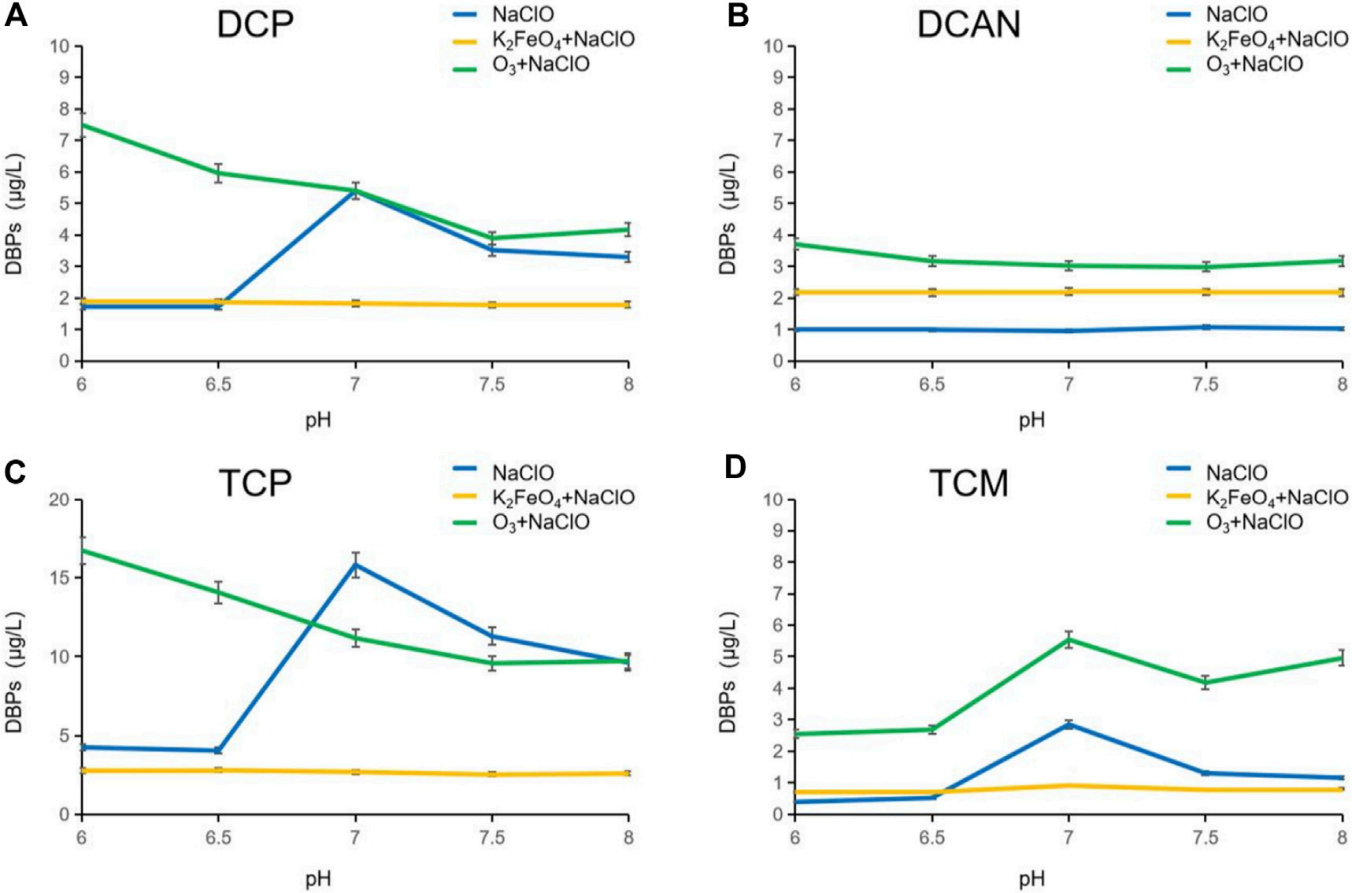
Influence of the pH value on DBPs formed after preoxidation of SMZ by K_2_FeO_4_ and O_3_.

The pH value greatly influenced the dissociation of SMZ and NaClO, which led to differences in the final degradation effect of the products as well as the types and production of DBPs. Research suggested that when the pH value of water is between 6 and 9, the chlorine would exist mainly as HClO and ClO^−^. In this study, the reaction temperature was controlled at 20°C, and the pH values varied from 6 to 8 (Deborde and Gunten, 2008). When the pH value was increased from 6 to 8, ClO^−^ became the main form of chlorine instead of HClO. This change in the existing form will greatly affect the oxidative degradation efficiency of NaClO. As shown in Figure 3, the number of DBPs formed in NaClO disinfection alone under neutral conditions increased significantly. DCAN was not obviously changed, and the poor denitrification effect of NaClO showed the selective attack characteristics of NaClO. In an acidic environment, chlorine in the solution exists mainly as HClO, with strong oxidation, and its ability to destroy SMZ molecules is much greater than that of the substitution reaction, so few DBPs were formed. In an alkaline environment, chlorine in the solution exists mainly as ClO^−^, the oxidation capacity of which was lower than HClO, so oxidation was weakened and the substitution reaction was enhanced. The intermediate fall off caused by SMZ oxidization is easy to be replaced by chlorine-containing groups to form CL-DBPs. The formation of DBPs was less affected by the pH value after preoxidation by K_2_FeO_4_, which may be due to the fact that the DBPs in the forms of HClO and ClO^−^ have no obvious selectivity toward the oxidation process of intermediates generated after preoxidation. The preoxidation effectively inhibited the production of DBPs. Compared with NaClO disinfection alone, preoxidation by O_3_ broke the energetic barriers of some reaction sites of SMZ, and the excess oxidation capacity in the subsequent chlorination reaction correspondingly destroyed more SMZ molecules and produced a large number of DBP precursors, resulting in the increase of DBPs.

### Influence of reaction temperature on DBPs formed after preoxidation of SMZ by K_2_FeO_4_ and O_3_

**FIGURE 4:**
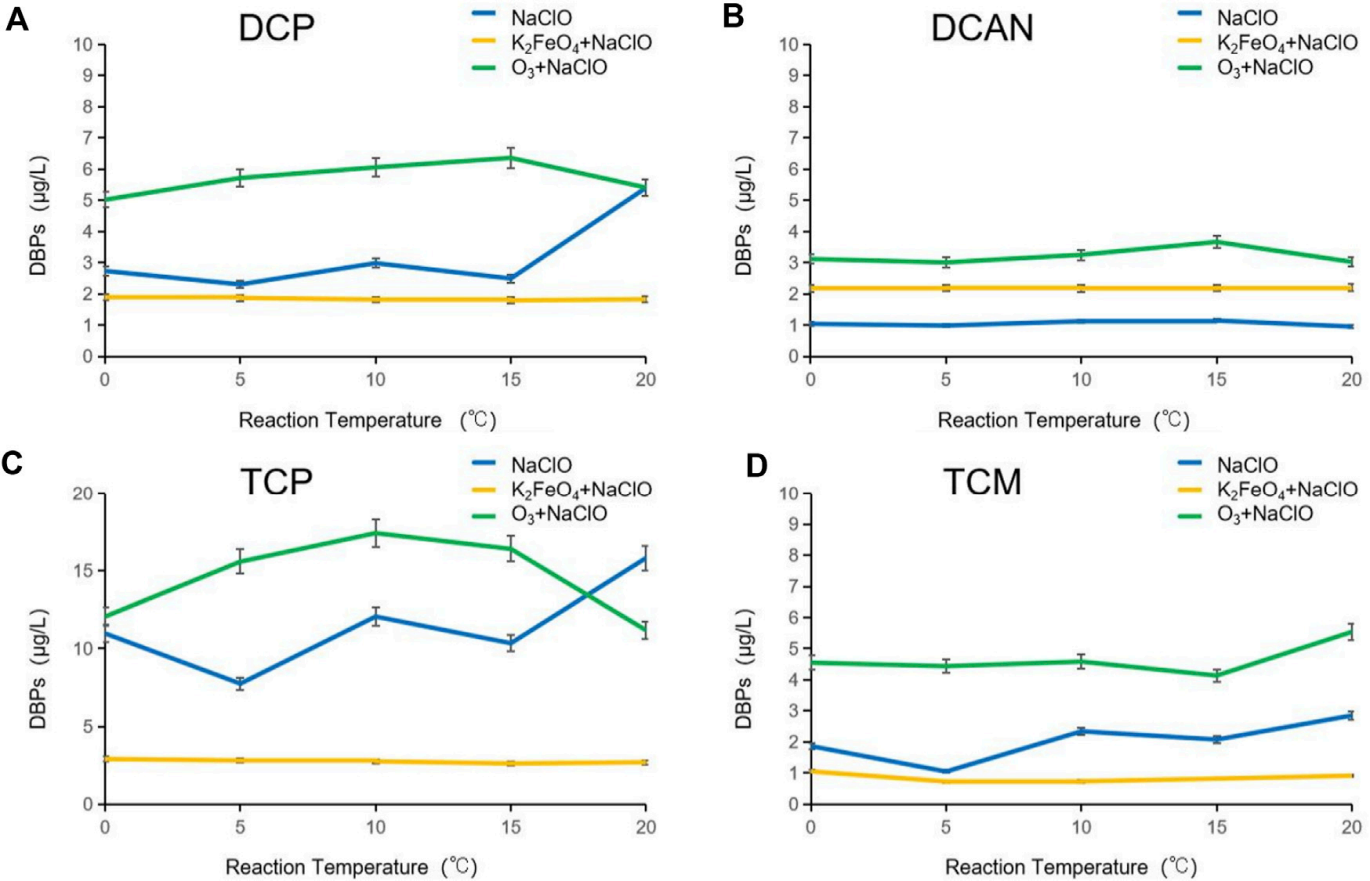
Influence of reaction temperature on DBPs formed after preoxidation of SMZ by K_2_FeO_4_ and O_3_.

The formation of DBPs after preoxidation of SMZ by K_2_FeO_4_ and O_3_ at different temperatures is shown in Figure 4. When disinfecting with NaClO alone, the increase in temperature obviously influenced the formation of DBPs. With the increase in temperature, the rise in the production of four kinds of DBPs fluctuated, with the change rate varying between 8.7 and 53.2%. After preoxidation by K_2_FeO_4_, the generation of four types of DBPs was not greatly affected by the increase in temperature, but with certain regularity, showing a trend of first decreasing and then increasing. The production of DCP, TCP, and TCM was significantly lower than that of NaClO disinfection alone, and the production of DCAN was about twice higher than that of NaClO disinfection alone. The change trend of DBPs after preoxidation by O_3_ was similar to that by K_2_FeO_4_, and the production of DCP, TCP, and DCAN showed a trend of first decreasing and then increasing. The formation of TCM showed an opposite trend, increasing after 15°C. This may be because the solubility of O_3_ was decreased, and the reaction effect was reduced with the increase in temperature, but the reaction activity of NaClO increased with the temperature. There is an inflection point at the temperature of 10–15°C, indicating that the reaction activity of O_3_ and NaClO reaches a relative equilibrium state and promotes the formation of DBPs.

### Influence of FA on DBPs formed after preoxidation of SMZ by K_2_FeO_4_ and O_3_

Humic acid widely exists in natural water and is an important part of the ecosystem. Fulvic acid (FA), a type of humic acid, usually accounts for more than 80% of the total amount of humic acid. It easily dissolves in water and cannot be easily removed by the conventional water treatment process. It is an important DBP precursor formed in the process of water disinfection. In order to explore the change of DBPs formed in SMZ chlorination after preoxidation by K_2_FeO_4_ and O_3_ in natural water, fulvic acid was used to simulate natural water to conduct the research.

**FIGURE 5:**
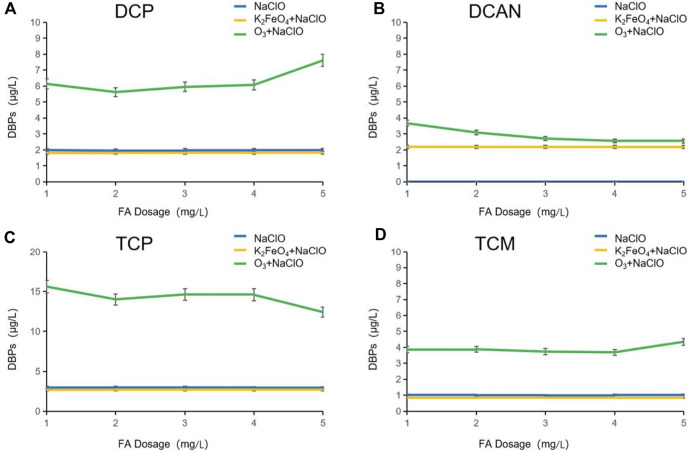
Influence of FA dosage on DBPs formed after preoxidation of SMZ by K_2_FeO_4_ and O_3_.

As shown in Figure 5, after NaClO disinfection alone, the dosage of FA was increased from 1 mg/L to 5 mg/L. In this period, no DCAN was formed, and the change rate of the other three DBPs was less than 5%. The production of DBPs produced after preoxidation with K_2_FeO_4_ was slightly lower than that disinfected by NaClO alone, and the difference lies in the formation of DCAN. After preoxidation with O_3_, it can be seen from the continuous decline of DCAN production that O_3_ has selectivity for FA degradation. The presence of FA inhibited O_3_ from attacking SMZ sulfonamide, therefore reducing the formation of N-DBPs. After preoxidation by O_3_, when the dosage of FA was increased from 1 mg/L to 2 mg/L, the production of all the other three DBPs decreased. When the dosage was increased from 2 mg/L to 4 mg/L, the production of DCP and TCP increased, while that of TCM decreased gradually. This may be due to the reason that the increase in FA dosage had inhibited the production of TCM by DBPs in the form of HOCl oxycodone. When the dosage was increased from 4 mg/L to 5 mg/L, the production of DCP and TCM increased, while the production of TCP decreased gradually, which may be caused by the inhibition of FA that consumed a large amount of HOCl and ClO^−^, and the conversion from DCP to TCP was weakened.

### Influence of iodide ions on DBPs formed after preoxidation of SMZ by K_2_FeO_4_ and O_3_

Supplementary Figure S1 shows the change in DBP formation after preoxidation in the presence of iodide ions. Only THMs were generated with chlorination alone. Supplementary Figure S2 shows the influence of KI concentration on preoxidation by K_2_FeO_4_ and O_3_. The production of I^−^DBPs increased with the increase of I^−^ concentration, and the production of Cl^−^ DBPs was reduced accordingly. This was because more HOI was formed at higher I^−^ concentration, which promoted the formation of I^−^ DBPS. After preoxidation by K_2_FeO_4_, the composition of DBPs changed greatly. In addition to the original TCP and TIM, DCP, DCIM, and TCP, etc., were also formed. With the increase in iodide ion concentration, it was observed that the production of TIM increased by 122.5%, while that of TCP decreased by 68.7%. This was because the oxidation of NaClO and HOI promoted the conversion of TCM to TIM. After preoxidation by O_3_, the oxidation of NaClO was stronger, and the production of DCP and TCP decreased continuously. The DBPs in the form of iodomethane and diiodomethane were oxidized by HOI to form TIM, and the production of TIM increased continuously.

### Influence of bromide ions on DBPs formed after preoxidation of SMZ by K_2_FeO_4_ and O_3_

Supplementary Figure S3 shows the change in DBP formation after preoxidation in the presence of bromide ions. Bromide ions in water samples promoted the formation of a variety of Br^−^ THMs and changed the distribution and production of DBPs. From Supplementary Figure S4, we can see that the production of Br^−^ DBPs increased with the increase of I^−^ concentration, and the production of Cl^−^ DBPs reduced accordingly. This was because more HOBr was formed at higher Br^−^ concentrations, which promoted the formation of Br^−^ DBPs (Hua et al., 2006). After preoxidation by K_2_FeO_4_, the composition and structure of DBP precursors may change greatly, resulting in a significant decrease in the total amount of DBPs produced by subsequent NaClO disinfection, and the production was not largely affected by Br^−^ concentration. The existence of Br^−^ promoted the formation of THMs and improved the Br^−^ distribution coefficient. The production of tribromomethane (TBM) was increased by 15.6%, while the production of TCP was decreased by 6.9%, which was mainly due to the oxidation effect of HOBr. In addition, the denitrification of SMZ by K_2_FeO_4_ promoted the formation of DCAN, which was converted into dibromoacetonitrile (DBAN) with stronger toxicity due to the effect of HOBr. BCAN only appeared after preoxidation by K_2_FeO_4_. After preoxidation by O_3_, the production of DBCM and DBAN increased by 41.3 and 281.1%, respectively, and the production of DBPs in the form of THMs decreased by 37.9%, which may be because the preoxidation of O_3_ destroyed more sulfonamide groups and the stripped nitrogen combined with HOBr to form DBAN. At the same time, preoxidation with O_3_ promoted the formation of alcohol from DBPs in the form of methane and reduced the formation of DBPs in the form of methane.

### Cytotoxicity and biological toxicity evaluation of DBPs formed by SMZ chlorination after preoxidation by K_2_FeO_4_ and O_3_

**FIGURE 6:**
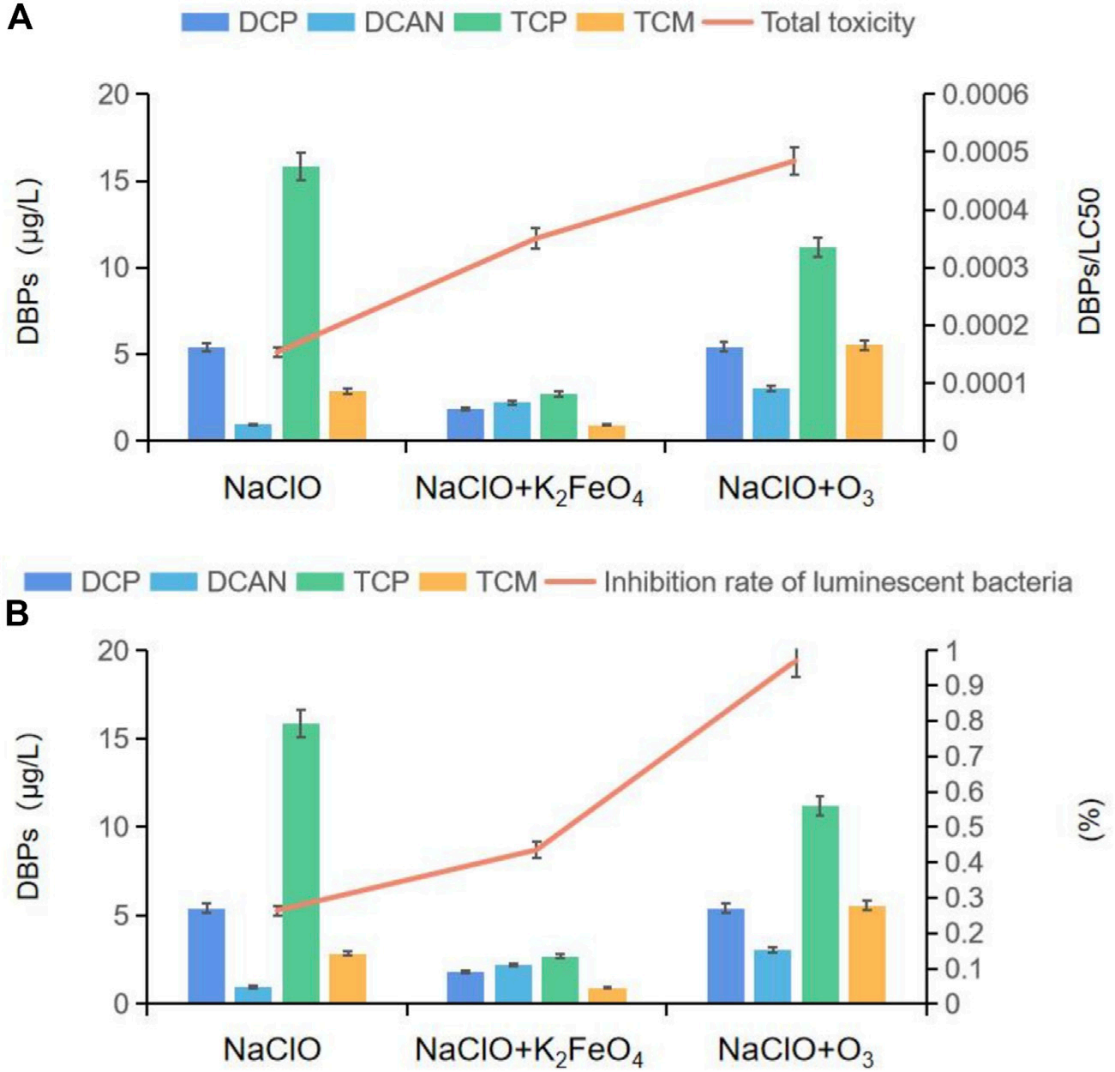
**(A)** Relation between the production of DBPs and cytotoxicity; **(B)** relation between the production of DBPs and biological toxicity.

Cytotoxicity was evaluated by dividing the molar concentration of DBP by its corresponding LC_50_ value. Four DBPs were detected after SMZ chlorination alone and SMZ chlorination after preoxidation by K_2_FeO_4_ and O_3_. As shown in Figure 6A, after preoxidation by K_2_FeO_4_, the production of DCP, TCP, and TCM decreased significantly, and the content of DCAN increased by 131.6%. After preoxidation by O_3_, the production of DCP and TCP decreased, but the production of TCM and DCAN increased by 94.2 and 217.8%, respectively. In the cytotoxicity calculation, the lower the LC_50_ value, the greater the calculated toxicity. Combined with the analysis of LC_50_ values of several DBPs assessed using the previously described method by Plewa et al. (Wagner, E. D. et al., 2017), the calculated cytotoxicity after preoxidation by K_2_FeO_4_ in this study was much lower than that by O_3_, and the cytotoxicity was reduced by 27.70%. In order to better prove that preoxidation by K_2_FeO_4_ was more effective than preoxidation by O_3_ in controlling the toxicity of DBPs formed in SMZ chlorination, a sensitive photoelectric measurement system was used to determine the influence of poisons on the luminous intensity of luminescent bacteria. The biological toxicity analysis results after two preoxidation methods are shown in Figure 6B. Two hours after chlorination of SMZ preoxidized with K_2_FeO_4_, the effective inhibition rate reached 43.56%; after preoxidation by O_3_, it was increased to 97.13%, and the inhibition rate was increased by 53.57%. Both toxicity evaluation methods showed that preoxidation by K_2_FeO_4_ was more effective than preoxidation by O_3_ in controlling the toxicity of DBPs formed after SMZ chlorination. However, we can also see from Figure 6 that both cytotoxicity and biotoxicity increased after preoxidation, but the values were still within the standard safety range (UNECE, 2022). The WHO “Guidelines for Drinking-Water Quality” clearly stipulate the concentration of various disinfection by-products (WHO, 2006) and are published on the US EPA website. The latest research results, “the maximum allowable concentration of pollutants in drinking-water quality standards,” also include disinfection by-product indicators (USEPA, 2009). In this study, the concentration of disinfection by-products after preoxidation was significantly reduced, and we believe that preoxidation is safe.

## Conclusion

This article studied the formation and toxicity of DBPs formed in SMZ chlorination with K_2_FeO_4_ and O_3_ as preoxidants. Under different dosages of NaClO, the preoxidation by O_3_ and K_2_FeO_4_ reduced the average formation of DBPs by 12.22 and 65.90%, respectively; preoxidation by K_2_FeO_4_ promoted the production of disinfection by-products by 3.02% under acidic conditions and 54.5% under alkaline conditions. The preoxidation by O_3_ promoted the formation of DBPs under both acidic and alkaline conditions. At different temperatures, the level of DBPs formed after preoxidation by K_2_FeO_4_ was lower than that by preoxidation by O_3_ and NaClO disinfection alone. Bromide and iodide ions make the components of DBPs formed after SMZ chlorination more complex. The cytotoxicity and biological toxicity of preoxidation by K_2_FeO_4_ were much lower than those by O_3_, which were reduced by 27.70 and 53.57%, respectively, but slightly higher than those of chlorination alone but still within the safe range. The degradation rate of SMZ after preoxidation by K_2_FeO_4_ was 8.41% higher than that of chlorination alone. In general, preoxidation by K_2_FeO_4_ has more obvious safety advantages for chlorination removal of SMZ than preoxidation by O_3_.

## Data Availability

The original contributions presented in the study are included in the article/Supplementary Material; further inquiries can be directed to the corresponding author.
