# Supplementary material for: Comparison of disinfection by-products formed by preoxidation of sulfamethazine by K2FeO4 and O3 and the influence on cytotoxicity and biological toxicity
Source: Front Chem. 2022 Aug 19;10:904867. doi: 10.3389/fchem.2022.904867 (PMC9437206; doi:10.3389/fchem.2022.904867)
Supplement: Supplementary file 1 [file DataSheet1.docx]

***Supplementary Material***

1. **Supplementary Figures**


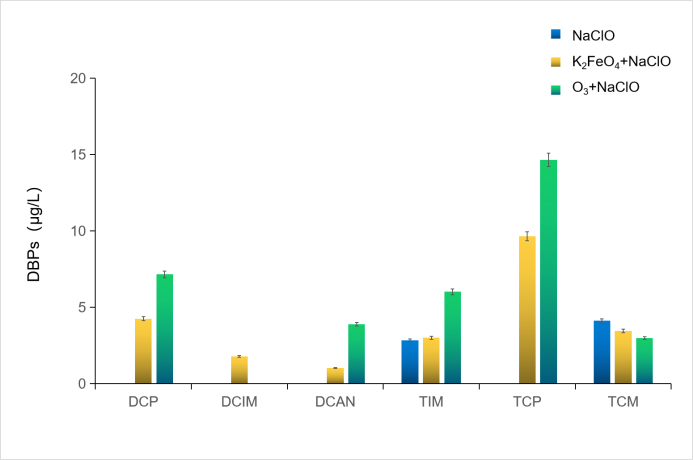


**Supplementary Figure 1.** Influence of I^-^ on DBPs formed after preoxidation of SMZ by K_2_FeO_4_ and O_3_


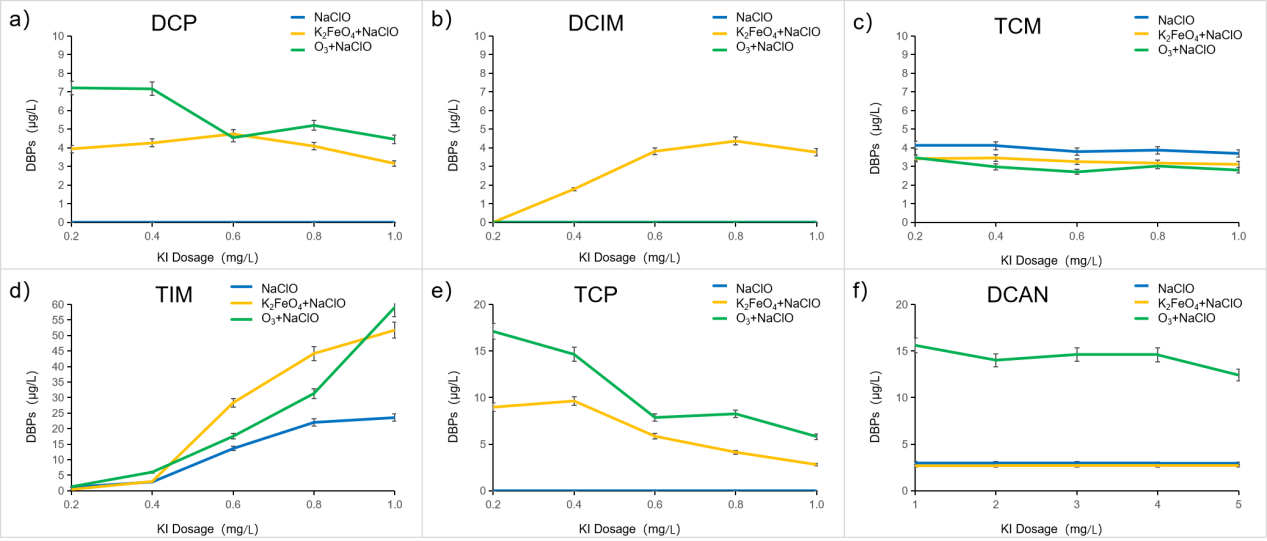


**Supplementary Figure 2.** Influence of KI dosage on DBPs formed after preoxidation of SMZ by K_2_FeO_4_ and O_3_


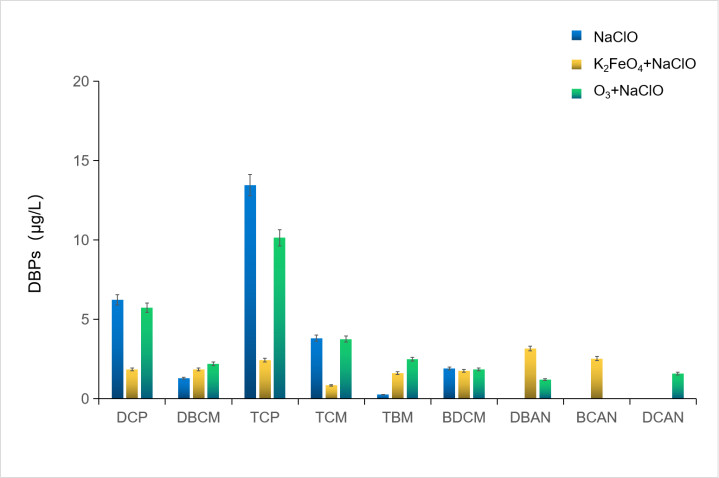


**Supplementary Figure 3.** Influence of Br^-^ on the change of DBP formation in SMZ chlorination after preoxidation by K_2_FeO_4_ and O_3_


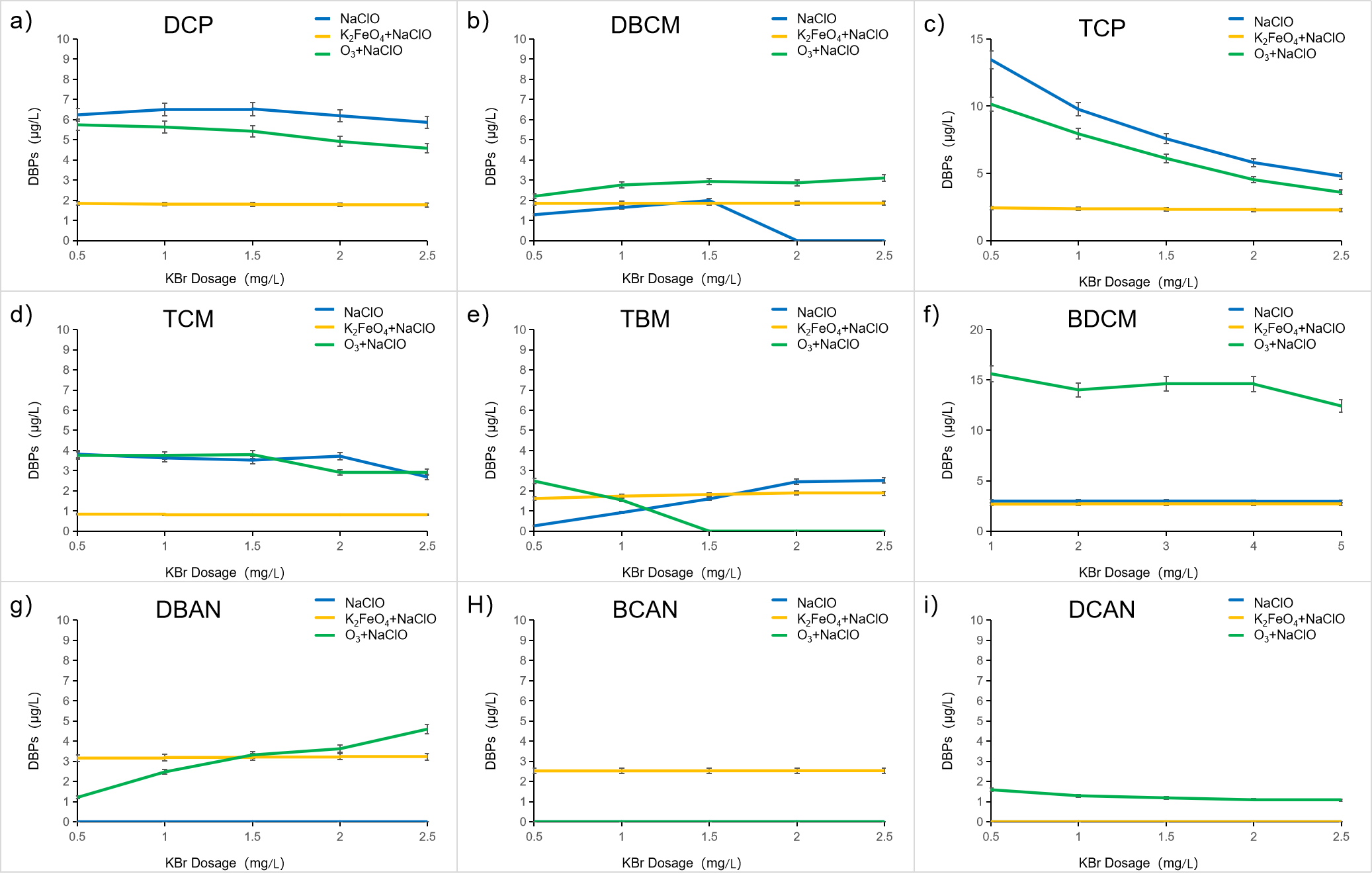


**Supplementary Figure 4.**Influence of KBr dosage on DBP formation after preoxidation of SMZ by K_2_FeO_4_ and O_3_
